# Design, implementation and analysis of a quality assurance process for Informed Consents using the DZHK registry TORCH-DZHK1 as an example

**DOI:** 10.1371/journal.pdig.0000798

**Published:** 2026-02-24

**Authors:** Dana Stahl, Katrin Leyh, Alexander Rudolph, Arne Blumentritt, Kerstin Weitmann, Monika Kraus, Johannes Trebing, Julia Hoffmann, Farbod Sedaghat-Hamedani, Benjamin Meder, Wolfgang Hoffmann

**Affiliations:** 1 Trusted Third Party of the University Medicine Greifswald, Ellernholzstraße, Greifswald, Germany; 2 DZHK (German Centre for Cardiovascular Research), Site Greifswald, Germany; 3 Institute for Community Medicine, Section Epidemiology of Health Care and Community Health, University Medicine Greifswald, Ellernholzstraße, Greifswald, Germany; 4 Institute of Epidemiology, Helmholtz Zentrum München, German Research Center for Environmental Health, Neuherberg, Germany; 5 DZHK, Site München, Germany; 6 Institute for Cardiomyopathies Heidelberg (CFH.), Department of Medicine Trial Unit, Clinic for Cardiology, Angiology and Pneumology of III, University Hospital Heidelberg, Heidelberg, Germany; 7 DZHK, Site Heidelberg/Mannheim, Heidelberg, Germany; 8 DZHK (German Centre for Cardiovascular Research), Main Office, Berlin, Germany; CHOP: The Children's Hospital of Philadelphia, UNITED STATES OF AMERICA

## Abstract

To collect sensitive patient data during clinical trials, the Informed Consent (IC) of the participants must be obtained beforehand. If the IC is not correct and complete, the document cannot be used to represent the will of the participant and will not be considered a legally valid document. However, few studies have examined the quality of the IC and the IC-quality found is unfortunately not satisfactory. The aim of this article is to describe the development of an IC quality assurance concept and to report the results of an evaluation using the example of a German Centre for Cardiovascular Research (DZHK) registry. All quality issues identified during the study were documented. These were aggregated into the quality indicators “Completeness”, “Consistency of Data”, “Correctness” and “Validity”. Of 2,453 ICs, 1,588 had at least one quality issue; 99.8% of them were resolved. In addition, training sessions were conducted with study staff to raise awareness of the importance of correct IC collection, including documentation, and to minimize quality issues. Our data exemplify that improvements in the recording of ICs by the study staff can be achieved. This evaluation shows the value and importance of continuous IC quality control.

## Author summary

In clinical research, informed consent is essential to ensure that participants voluntarily agree to the use of their personal and medical data. Only when consent forms are complete and accurate can they truly reflect a participant’s decision and meet legal requirements. Despite its importance, the overall quality of informed consent documentation has rarely been studied and is often found to be insufficient. In this work, we describe the development of a structured approach to improve and monitor the quality of informed consent forms using a large cardiovascular research registry in Germany as an example. By systematically reviewing consent documents, we were able to identify common problems, such as missing information, inconsistencies, or invalid entries. Importantly, nearly all identified issues could be successfully corrected. In addition, targeted training sessions were provided to study staff to increase awareness of the importance of proper consent documentation and to prevent future errors.

Our findings show that informed consent quality can be substantially improved through continuous quality control and education. This approach helps protect the rights of study participants, strengthens the legal reliability of consent documents, and supports the overall integrity of clinical research.

## Introduction

According to the European General Data Protection Regulation (EU-GDPR), all person-related sensitive data throughout Europe must be collected on the basis of Informed Consent (IC) (1). The IC must be filled in completely and correctly in order to represent the participant’s will as a legally valid document. ‘Complete’ means, among other things, the presence of the participant’s dated signature, and ‘correct’ means the unambiguous selection of optional modules [1,2]. In multicentre and large clinical trials, it is advisable to manage ICs as electronically and centrally as possible [2–3]. This is especially true for research collaborations that want to use data across studies for further research projects.

According to Good Clinical Practice (GCP) [1], monitoring of study data should contribute to safeguarding patients’ rights. However, in most cases only random samples of study data are examined, which means that only subsets of individual medical data sets including the IC s are quality assured. The Declaration of Helsinki [4] states that each participant in a clinical trial must sign an IC. This ethical requirement is further specified in Article 9 of the GDPR [5], which defines the processing of special categories of personal data like data concerning health on the basis of an IC. Personal data concerning health including person-identifying data belong to these special categories as sensitive data and, therefore, require special protection.

A structured literature search on data quality measures in registries (search terms “patient registry”, “patient registries”, “quality”, “data quality”, “quality control”) in PubMed revealed that source data verification is most of the time performed as classical monitoring [6–20], which is mostly based on samples due to cost and time constraints [8,9,13,21]. Using this method, only a few individual data sets, both ICs and participants’ medical data, are sampled for quality assurance [22].

**Fig 1:**
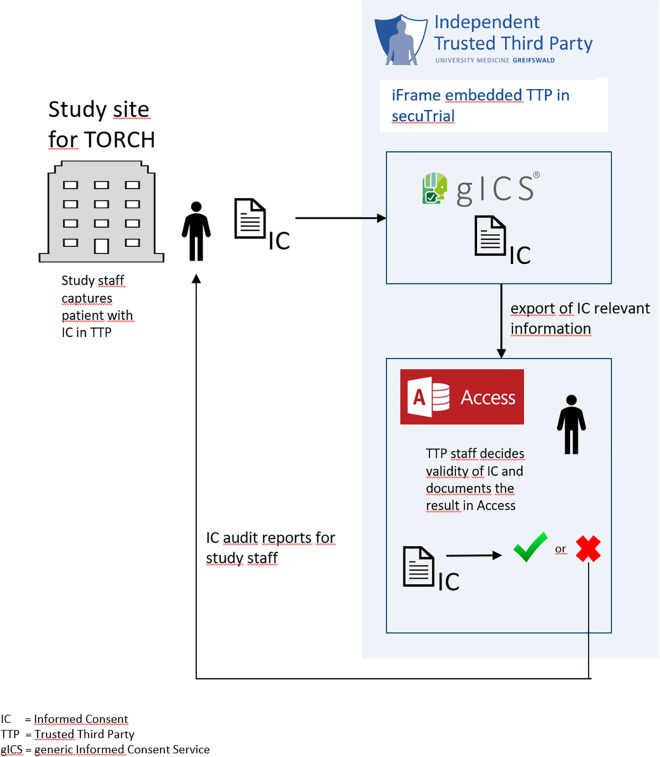
Process of data entry in TTP; including gICS and quality assurance process.

According to the TMF Data Protection Guide 2.0 [23], identifying data of participants, which includes ICs, should in principle be processed separately from medical data in medical research, to prevent re-identification of individual participants as much as possible. For this purpose, a Trusted Third Party (TTP) [24] was established as part of the central DZHK research platform. The TTP electronically manages ICs using the generic Informed Consent Service (gICS) [3] software tool. ICs are primarily collected on paper. As seen in Fig 1, in DZHK studies, ICs are entered into an electronic Case Report Form (eCRF) of the Trusted Third Party based on the completed paper-based form. The paper-based IC is then scanned and attached to the electronically entered IC as a scan. All of these processing steps are supported by gICS, a special tool for managing ICs [3].

Vogele et al. [25] have shown in ICs for computed tomography of four clinics that approximately 18% of paper ICs are not filled out completely. If the signature is missing, the respective IC, for example, is not valid. This indicates that the quality of hand-filled ICs can be an issue. Furthermore, the likelihood that discrepancies may occur between an eCRF of the IC and the (scan of) paper-based IC due to manual entry can further increase the rate of quality issues. To assure valid ICs requires a quality assurance process. To our knowledge, there is no published best-practice example for this form of quality assurance.

To ensure the quality of ICs filled in on paper and, subsequently, entered into an electronical management system, a quality assurance concept was created, implemented, and evaluated on the basis of predefined quality indicators of Nonnemacher et al. [22]. This paper describes the IC quality assurance concept and the outcomes of the evaluation using the example of a DZHK registry, TranslatiOnal Registry for CardiomyopatHies (TORCH-DZHK1). As mentioned above, this publication might one of the first full-cohort IC quality assurance (QA) studies, as most others rely on sampling.

## Methods

### Ethics statement

The study TranslatiOnal Registry of CardiomyopatHies (TORCH-DZHK1) was proven in 2014 by Ethics Committee of the Medical Faculty of Heidelberg University for the main study centre and followed by a few amendments about the duration of the study. TORCH was continued as part of a follow-up project called TORCHplus from 2021. Participants had to voluntarily consent to participate in the study. The study team had to ensure that ethical and legal requirements were met.

TORCH-DZHK1 was the first national registry in the DZHK infrastructure. Multicentric data collection included Informed Consents, and biomaterial from 2,300 participants with myocardial diseases (cardiomyopathy) and a standardized eCRF for comprehensive patient characterization in a harmonized manner across all participating sites [26]. Recruitment started in December 2014 and was completed in January 2018. The goal is to deliver standardized and harmonized data and biomaterials optimized for a broad scientific use to the research community.

### Development of a quality assurance (QA) concept

In coordination with the ethics coordination project of the DZHK [27], three TORCH-DZHK1 IC versions (as of: 20.07.2021) were necessary due to changes in the study protocol and respective processes. All versions were voted positively by the ethics committees of the participating study centres, and were technically implemented within the TTP. Medical data have been quality assured as part of a comprehensive monitoring process during data collection [28]. Quality assurance of the identifying data has been performed in parallel to the project’s duration. This work focuses exclusively on the identifying data of TORCH-DZHK1 participants and their ICs.

A structured literature search focused on ICs using PubMed with the search terms: “monitoring”, “quality assurance”, “informed consent”, “clinical trials”, “data”, “research”, “GDPR, and “EU” was performed on 01/07/2020. No relevant results emerged except for a study by Vogele et al. [25], who only assessed completeness and readability of handwritten remarks.

As quality indicators for this work, the indicators “completeness” and “consistency of registry data with original data related to observation units” as “correctness” were selected from Nonnemacher et al. [22] (quality indicator IDs TMF-1046, TMF-1045). Furthermore, based on the above mentioned GCP and EU-GDPR, an additional indicator “validity of consent” was defined by the TTP. For example, for “completeness” [22] it is checked whether all data have been recorded or all pages of an IC scan are available. For the indicator “correspondence of the register data with the original data related to observation units” [22] it is verified whether the ICs recorded on paper equivalent to those of the electronically recorded ICs. To determine the indicator “validity of consent” [1,5,4], ICs are reviewed to ensure that there are both, the signatures of the participant and the physician, on each IC and both are appropriately dated.

For the determination of the indicators and the related development of the quality assurance concept, workshops were held with the study’s coordinators, staff, and monitor. In several webinars, best practices in quality assurance of ICs were agreed upon, which helped to specify the conceptual design.

Quality assurance was conducted using the software solution gICS [3]. The gICS provides a web interface for managing ICs, revocations, and study exclusions. Among other options, the user can view a) which ICs have been created electronically for a participant and b) can verify that a scan of the paper document has been uploaded for each participant and c) that both are complete and accurate. A reporting and feedback structure was also developed as part of the concept.

### Data analysis

At the TTP, a weekly quality review of newly created ICs was conducted. All newly generated ICs were assessed by the responsible staff member in accordance with the established QA concept. Relevant findings were systematically documented and communicated back to the study personnel.

A frequency analysis for TORCH was performed based on the issue types defined above in order to assess whether certain types of errors occurred more frequently than others. Recurrently identified issues were further analyzed to derive potential needs for technical adjustments or targeted training measures.

An assessment of inter-rater reliability was not feasible due to the substantial manual effort already required to review ICs for approximately 2,300 participants. The QA concept was intentionally designed to be sufficiently specific to allow implementation by a single reviewer. In cases of ambiguity, the responsible staff member could consult with colleagues or study personnel to reach a valid review decision. The concept is structured to define explicit criteria for issue categorization (see Table 4), thereby enabling new staff members to independently achieve review results consistent with those of experienced reviewers.

## Results

For the 2,300 recruited TORCH-DZHK1 participants, a total of 2,453 ICs were recorded by study staff and quality checked by the Trusted Third Party. For these ICs, 1,588 quality issues were recorded by the Trusted Third Party from December 2014 to February 2018.

Fig 1 shows that April 2016 has a clear peak in ICs with errors and that this number dropped considerably over the next year until 2017 and remained almost constant at a low level.

One IC can have more than one finding. 1,307 ICs (53,28%) were initially correct and had no quality issues. 702 ICs had only one issue, 274 ICs included two issues and 170 ICs had more than two issues with most (N = 94) having three inconsistencies. The maximum was eight different reported issues in one IC.

As a result of this quality assurance concept, all quality issues identified regarding IC quality during the course of the study were documented in a Microsoft Access (see Fig 1) database created specifically for IC quality assurance. Detailed reports (so-called IC audit reports) were generated reporting these anomalies, which supported the study staff in dealing with the identified anomalies. The IC audit reports were made available to study staff as PDFs via an encrypted ticket system. Furthermore, aggregated reports were created for the study coordinators (so-called feedback reports), in which the quality of the ICs and the processing status of the IC errors, among other things, are presented across all study centres of a study. These feedback reports serve the study coordinators as a holistic overview of the data quality of the ICs in their study (Table 1).

**Table 1:** Assignment of quality issues to categories for the quality indicator completeness.

Categories	possible quality issues
Pseudonym	• item missing
Gender	• item missing
Date & place of signature (physician & participant)	date of signature (physician or participant) missing place of signature (physician or participant) missing date & place (physician or participant) missing
Name of participant	• item missing
Date & place of birth of participant	place of birth missing date of birth missing place of birth & date of birth missing
name of physician	• item missing

Therefore, the quality assurance process was developed as follows: All ICs were checked and may have several inconsistencies during quality assurance. These quality issues were assigned to specific categories, which in turn were assigned to a quality indicator according to Nonnemacher et.al. [22]. An IC can have several findings per category at the same time, e.g., “date and place are swapped” and “place not legible”, each are counted individually in the errors statistics. An overview of all categories by indicator and which action is required to fix the errors can be found in Table 4.

### Operationalisation of quality indicator ‘completeness’

**Fig 2:**
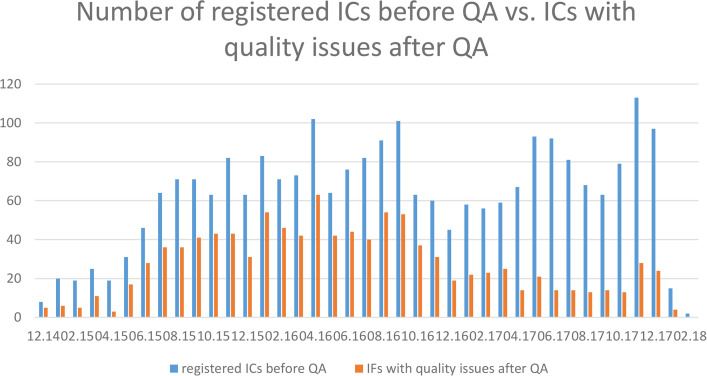
Comparison of recorded ICs with recorded quality issues in TORCH-DZHK1 per month for the period Dec. 2014 to Feb. 2018.

It can be seen (see Fig 2) that the most frequent trigger for this indicator was that the pseudonym was not stated on the IC. This pseudonym is important for the Trusted Third Party in order to be able to track whether the IC scan was uploaded for the correct person. If this is not the case, the study centre will be asked to make corrections (Table 2).

**Table 2:** Assignment of issues to categories for the quality indicator correctness.

Categories	Possible quality issues
Pseudonym	not legible discrepancies (item not stated In dedicated area of the IC, wrong pseudonym)
Gender	not stated In specific item area discrepancies (inconsistent gender selection)
Date & place of signature (physician & participant) Note: Auditor has to decide for one of the quality issues per category	date not stated In dedicated area of the IC place not stated in dedicated area of the IC date & place not stated in dedicated areas of IC date & place are documented in the same area date & place are swapped date incomplete date not legible place not legible date & place not legible
Name of participant	not stated in dedicated area of the IC not legible information missing partly discrepancies (inconsistencies between name documented on paper-based informed consents and digitally in TTP)
Date & place of birth of participant	place of birth not stated in dedicated area of IC date of birth not stated in dedicated area of IC date & place of birth not stated in dedicated area of IC date & place of birth were swapped place of birth not legible date of birth not legible discrepancies (inconsistencies between date or place of birth documented on paper-based informed consents and digitally in TTP)
Signature of physician or participant	not legible (e.g., cross instead of signature) not stated in dedicated area of the IC
Name of physician	• not stated in dedicated area of the IC

### Quality indicator ‘correctness’

**Fig 3:**
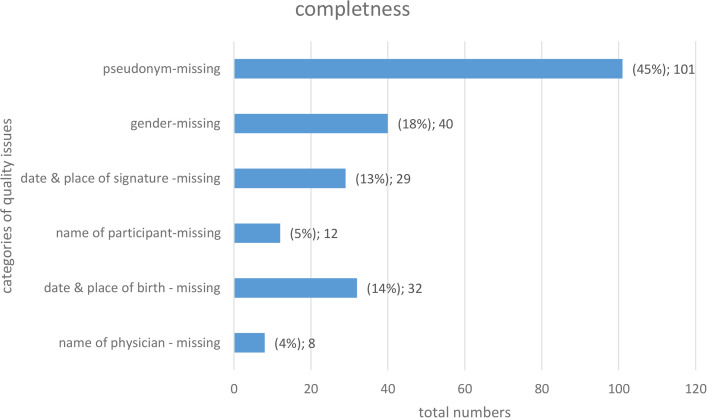
Quality issues for the quality indicator “completeness“.

Fig 3 shows that the most frequent errors occur when filling in the name of a participant, as well as the date and place of signature. This can lead to record linkage difficulties and make it difficult or impossible to identify the participant clearly, in order to validly map the participant’s will (Table 3).

**Table 3:** Assignment of anomalies to categories for the quality indicator validity of consent.

Categories	possible quality issues
Signature of physician and participant	• one or both items missing
Optional modules	discrepancies (crosses not unambiguously set) discrepancies between IC-scan and digital representation
IC-scan	not legible (scan too light or too dark) item missing incomplete (e.g., missing pages) wrong IC version IC Scan of another participant uploaded

### Quality indicator ‘validity of consent‘

**Fig 4:**
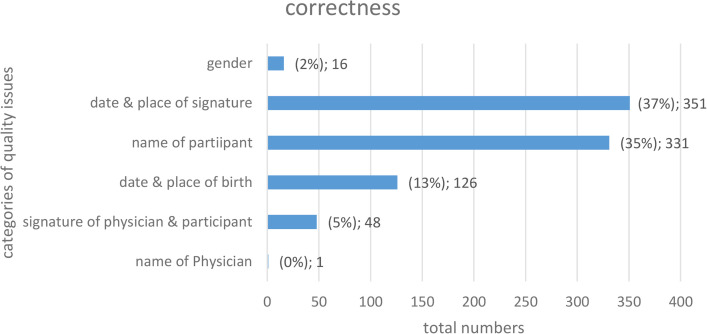
Quality issues for the quality indicator “correctness“.

Fig 4 shows that, most frequently, IC scans were not uploaded correctly. These are either a) incompletely scanned ICs or b) completely missing scans of the paper-based consents.

**Fig 5:**
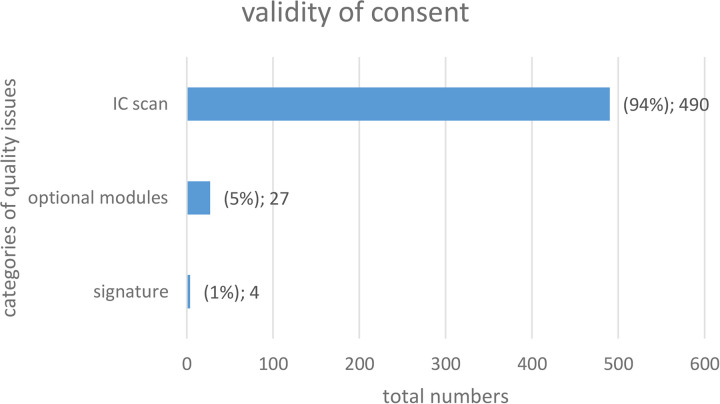
Quality issues for the quality indicator “validity of consent“.

The quality assurance reports are sent to staff from the recruiting study centres, which are asked to correct any unclear, inconsistent or erroneous data (Fig 5).

As of February 2018, at the end of the recruitment phase, two unresolved IC-related quality issues remained:

(1) missing IC scan: In the absence of an IC scan, the validity of the participant’s consent could not be verified. Consequently, the participant and all associated data were deleted by the Trusted Third Party, and corresponding deletion instructions were issued to all other involved entities. As a result, the respective dataset is permanently unavailable for research purposes.
(2) Discrepancies between the optional module selections documented in the IC scan and those recorded in the corresponding digital IC:

In cases involving discrepancies in module selection, the paper-based IC was considered legally binding. Accordingly, the digital IC was corrected to accurately reflect the paper-based documentation, thereby allowing the participant’s data to remain available for research use.

### Quality assurance concept based on the quality indicators

As one part of the development of a coherent quality assurance concept, the quality issues were ranked on the basis of their need for action. As a second step, the practicality of need for actions (study staff needs to correct IC) are considered.

Inconsistencies are generally excluded from a need for action, if a) information is unmistakable but not in the designated field; or b) unmistakable information, such as date and place, has been interchanged. Additionally, the place of signature is required by GCP, but is not required for validity of consent. Therefore, anomalies related to this field are also not considered actionable by the TTP.

Thus, for the quality indicator completeness, all fields must be filled in except for the place of signature. For the quality indicator correctness, all findings that cannot be unambiguously assigned to an IC data entry field or to the digitally recorded data in the TTP database by manual editing must be corrected.

For the quality indicator validity of consent, every errors requires action, since a valid IC with correct representation of the participant’s decisions is mandatory. Table 4 describes all three categories with their possible quality issues and whether those require action or not according to this ranking.

**Table 4:** Need for action by indicator, category and quality issues.

categories	possible quality issues	action required?
Completeness		decide on a case-by-case basis
Pseudonym	• item missing	yes
Date & place of signature (physician or participant)	• date of signature of physician or participant missing	yes
	• date & place of signature of physician or participant missing	yes
Name Of Participant	• item missing	yes
Date Of Birth & Place of birth	• date of birth missing	yes
	• place of birth missing	no
	• date & place of birth missing	yes
Name of physician	• item missing	yes
Correctness		decide on a case-by-case basis
Pseudonym	• not legible	yes
	• discrepancies (not in dedicated field, wrong pseudonym)	yes
Date & place (physician or participant)	• Date not legible	yes
	• Date incomplete	decide on a case-by-case basis
	• Date & place not legible	yes
Name of participant	• Not legible	yes
	• Missing partly	yes
	• Discrepancies (different name was digitally documented in TTP)	yes
Date of birth & place of birth	• date of birth not legible	yes
	• Discrepancies (different data was digitally documented in TTP)	yes
Signature of physician or participant	• not legible (e.g., cross instead of signature)	yes
	• is not in dedicated field	decide on a case-by-case basis
Validity of consent		yes
Signature	• item missing	yes
Optional modules	• discrepancies (crosses not clearly set)	yes
	• discrepancies between IC-scan and digital representation	yes
IC-Scan	• not legible (scanned too light or dark)	yes
	• missing	yes
	• incomplete	yes
	• wrong version or variant	yes
	• IC Scan of another participant uploaded	yes

IC quality assurance in the TORCH-DZHK1 registry was based on this general quality assurance concept and evaluations were performed once a week. All detected quality issues were recorded in the described Access database for TORCH quality assurance. Once a month, the above-mentioned IC audit reports were generated from the collected findings that require action and were distributed to the study centres to initiate the corrections. In addition, aggregated reports were generated including all issues and incorporated into the monthly feedback reports. These were sent to the study coordinator of TORCH-DZHK1.

### Other quality-enhancing measures and site-specific issues

From December 2014 to February 2018, 25 training sessions were conducted with study staff to raise awareness of the importance of correct IC collection, including documentation, and to minimize IC quality issues. These were offered both online, and on-site as group or one-on-one training. In addition, telephone support is available for any procedural and technical questions.

**Fig 6:**
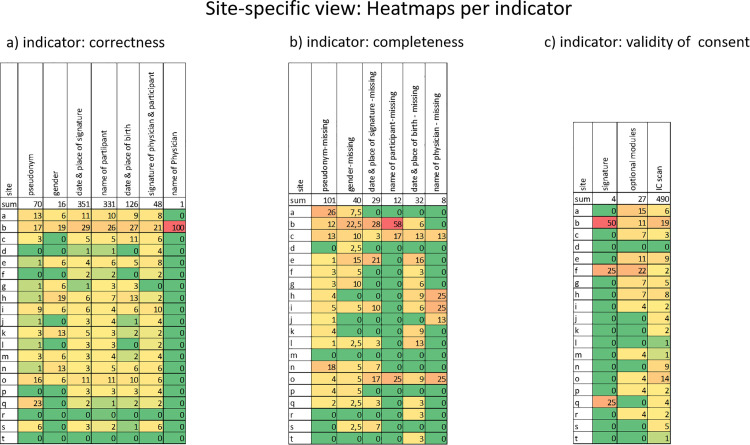
Heatmaps for a site-specific view of IC errors. The total row shows the number of cases that occurred. The heat map fields contain the percentage of IC errors per category. The site names have been pseudonymized. The green fields are to be assessed positively, as these sites have demonstrated a low to negligible error rate. The red fields, by contrast, indicate the sites with the highest proportion of errors.

Furthermore, variability was observed among the participating sites with respect to the frequency of errors. Fig 6 illustrates the distribution of errors by indicator, presented as heat maps showing each indicator’s percentage contribution to the respective total error category.

## Discussion

Quality issues of a IC mean that the IC may not be a legally sound representation of the study participant’s will. Accurate processing of the participant’s will is essential for conducting a study, data collection, analysis, and subsequent data usage. The quality check of the ICs revealed that learning effects and improvements in the recording of ICs by study personnel are discernible (Fig 1). However, the number of errors is subject to fluctuations in study staff and is permanently greater than 0 over the duration of the study period. This shows both the necessity and the efficacy of continuous quality control.

The low level of inconsistencies towards the end of the study indicates the overall success of the quality assurance concept. Fluctuations may be due to personnel changes in the study centres. The TTP cannot determine with certainty which registered staff members were involved in the study over its duration. However, available data suggest a staff turnover rate of approximately 30%.Almost all employees are working on multiple studies. Furthermore, new quality issues may result from errors made while correcting findings. In addition to the initial training and the monthly IC audit reports, the need for quality management and continuous follow-up training became apparent due to these fluctuations.

At the beginning of quality assurance in January 2015, it was assumed that after a short settling phase, checking random samples of ICs would be adequate. Source data verification is usually limited to random samples due to the extensive effort associated with this measure in quality assurance. However, the continuously high error rate, the fact that an improvement in quality could only be seen after several years and to a limited extent, excluded the restriction of IC inspections to random samples. Our results show that continuous quality assurance measures are essential to permanently ensure that the participant’s will is appropriately considered in all data processing and that there is no loss of data for research due to faulty ICs.

IC audit reports were initiated January 2016. Since then, the concept of IC quality assurance has been adapted over time. For example, some centres place patient stickers on the ICs instead of preparing the forms by hand. Because these stickers are generated from hospital information systems, the Trusted Third Party can assume that the information on the stickers is correct. However, the study centre and staff are responsible for making sure that they provide the correct participant’s IC and information when using participant label stickers. The IC fields were either completed in addition to the sticker or not completed at all if the study staff member used the sticker instead of making entries. Even if the information may not have been in the designated field, this was not considered as an error when using participant label stickers.

In the case of the signatures of participants and physicians, it was initially assumed that approximately the name of the respective person must be recognizable in order to determine whether the correct person also signed. This verification turned out to be neither feasible nor practical. This is in accordance with experiences of other large multicentric projects such as the German National Cohort [29] or the Baltic Fracture Competence Centre (BFCC) [30], e.g., a valid signature does not have to be legible.

The recipient of IC audit reports was initially the study director. The main reason for this rather centralised communication was that the Trusted Third Party did not have knowledge of which individual study staff employees were involved in collecting data at the centres. The study director was responsible for forwarding the IC review reports to the centres for processing and IC correction. To ensure the fastest possible processing this process was changed in 2017: Since then, with every new recruiting centre, a responsible person is named to the Trusted Third Party. This person receives and processes the IC audit reports. The procedure has proven to be very effective, because since then the quality issues listed in the reports are usually continuously processed and remedied.

While previous publications have primarily focused on specific samples, the present work provides an overview not only of the state of IC quality across an entire cohort but also of its temporal development over the course of the study. Each study should implement comprehensive quality assurance of its data. In addition to automated plausibility checks—which are supported by systems such as secuTrial [31], RedCap [32], or OpenClinica [33] for eCRF entry—our approach extends beyond these measures by systematically evaluating paper-based documents against predefined quality criteria.

To further automate these verification processes, a pilot implementation of AI-assisted quality assurance (state of the art as of 2025) could be considered, thereby minimizing the number of issues requiring manual review by TTP personnel. The QA concept was developed with reference to procedures established in secuTrial and RedCap. However, these procedures are not intended for implementation within gICS, as the goal is to achieve complete electronic capture of ICs in the future, as described below. Additionally, Optical Character Recognition (OCR) could be employed for verifying checkbox selections as well as handwritten pseudonyms and names. The gICS software engineering team is currently evaluating whether this represents a practical and time-efficient enhancement.

Another relevant consideration concerns the legibility of participants’ handwriting. Cardiology studies frequently involve older male participants. No definitive conclusions can be drawn regarding participants’ educational backgrounds. Handwriting quality varied considerably among individuals, and acute health conditions or pre-procedural anxiety may have further influenced legibility. At the time of data collection, these factors could not be investigated in greater detail. Such assessments would need to be conducted by study staff, who typically have limited resources for this type of inquiry. In summary, the complete electronic capture of ICs eliminates the need for TTP personnel to perform handwriting recognition, thereby simplifying and streamlining the QA process.

In recent years, the Trusted Third Party has developed the possibility of recording ICs in a legally secure and completely digital manner. SignPads or tablets can be used for this purpose, which support both the selection of optional IC modules and the signatures completely digitally. Thus, a transfer of handwritten data into IT systems would become obsolete and quality assurance could be reduced to a minimum. Consequently, this means that the check would then only include the presence of the signatures, a validation of the date of birth, and, if necessary, the correct IC version or IC variant. This digitalisation will save resources and create opportunities for further development. In the future, this system should be used primarily wherever technical conditions of study centres permit.

## Conclusion

This paper describes the development of a quality assurance system, whose feasibility has been adapted to the reality of everyday clinical recruitment practice in a multitude of study centres. In a practice test the concept proved applicable for the TORCH-DZHK1 registry.

Systematic quality assurance measures of ICs support an ethically and legally sound administration and mapping of the participant’s will, throughout the life cycle of a study. The developed routines are fully established in the DZHK research platform. They can readily be adapted to other central data management systems. This enables central participant management within the framework of research collaborations and supports sponsors in their responsibility for correct participant management.

However, overall, the permanent improvement of IC quality in the TORCH-DZHK1 registry could only be determined to a limited extent, as the same errors reappeared after a certain period of time. Mainly, the uploading of the IC scans was forgotten, which in the future can be solved by technical optimization of the process. Using tablets capturing ICs completely digitally will offer a safer alternative that also conserves resources by reducing sources of error.

Irrespective of the technical platform, maintenance of a high level of quality requires regular training. Feedback reports support the day-to-day management and should be provided throughout the duration of the study.

The quality assurance concept presented here will be further evaluated in the coming years for its practical suitability in other DZHK studies. Where necessary, it will be expanded to cover specific requirements that arise within the framework of the DZHK and other research institutions conducting complex clinical studies. The goal is to further optimize this concept as a best practice model for IC quality assurance in clinical trials and registries.

The COVID-19 pandemic has led to increased political impetus to further digitalize research processes. Since 2021, many new initiatives—such as the Network of University Medicine in Germany—that collaborate with the TTP have made extensive use of tablet-based IC documentation [34]. Moreover, gICS has already been integrated into a variety of technical systems for data capture in research settings. These include secuTrial (interActiveSystems), RedCap (Vanderbilt University), Meierhofer M-KIS, TrialComplete (T-Systems), and CentraXX (Kairos).
